# Interventions to improve school-based eye-care services in low- and middle-income countries: a systematic review

**DOI:** 10.2471/BLT.18.212332

**Published:** 2018-08-27

**Authors:** Anthea M Burnett, Aryati Yashadhana, Ling Lee, Nina Serova, Daveena Brain, Kovin Naidoo

**Affiliations:** a Brien Holden Vision Institute, Level 4, North Wing, RMB, Gate 14, Barker St, University of New South Wales, Sydney 2052, Australia.

## Abstract

**Objective:**

To review interventions improving eye-care services for schoolchildren in low- and middle-income countries.

**Methods:**

We searched online databases (CINAHL, Embase®, ERIC, MEDLINE®, ProQuest, PubMed® and Web of Science^TM^) for articles published between January 2000 and May 2018. Eligible studies evaluated the delivery of school-based eye-care programmes, reporting results in terms of spectacle compliance rates, quality of screening or attitude changes. We considered studies to be ineligible if no follow-up data were reported. Two authors screened titles, abstracts and full-text articles, and we extracted data from eligible full-text articles using the availability, accessibility, acceptability and quality rights-based conceptual framework.

**Findings:**

Of 24 559 publications screened, 48 articles from 13 countries met the inclusion criteria. Factors involved in the successful provision of school-based eye-care interventions included communication between health services and schools, the willingness of schools to schedule sufficient time, and the support of principals, staff and parents. Several studies found that where the numbers of eye-care specialists are insufficient, training teachers in vision screening enables the provision of a good-quality and cost–effective service. As well as the cost of spectacles, barriers to seeking eye-care included poor literacy, misconceptions and lack of eye health knowledge among parents.

**Conclusion:**

The provision of school-based eye-care programmes has great potential to reduce ocular morbidity and developmental delays caused by childhood vision impairment and blindness. Policy-based support, while also attempting to reduce misconceptions and stigma among children and their parents, is crucial for continued access.

## Résumé

**Objectif:**

Examiner les interventions permettant d’améliorer les services d'ophtalmologie pour les enfants scolarisés dans les pays à revenu faible et intermédiaire.

**Méthodes:**

Nous avons recherché dans des bases de données en ligne (CINAHL, Embase®, ERIC, MEDLINE®, ProQuest, PubMed® et Web of Science^TM^) des articles publiés entre janvier 2000 et mai 2018. Les études admissibles évaluaient la mise en œuvre de programmes d'ophtalmologie en milieu scolaire et en présentaient les résultats en termes de taux de suivi des recommandations, de qualité du dépistage ou de changements d'attitude. Nous avons considéré comme non admissibles les études qui ne comportaient pas de données de suivi. Deux auteurs ont parcouru des titres, des résumés et des articles intégraux, et nous avons extrait des données des articles intégraux admissibles selon le cadre conceptuel de disponibilité, d'accessibilité, d'acceptabilité et de qualité fondé sur les droits.

**Résultats:**

Sur les 24 559 publications examinées, 48 articles provenant de 13 pays remplissaient les critères d'inclusion. Les facteurs entrant en jeu dans la réussite des interventions d'ophtalmologie en milieu scolaire étaient la communication entre les services de santé et les établissements scolaires, la volonté de ces derniers d'y accorder suffisamment de temps, et le soutien des chefs d'établissement, du personnel et des parents. Plusieurs études ont révélé que lorsque le nombre de spécialistes en ophtalmologie est insuffisant, former les enseignants au dépistage des troubles visuels permet d'offrir un service économique et de bonne qualité. Outre le coût des lunettes, un faible taux d'alphabétisation, des idées fausses et un manque de connaissances des parents en matière de santé oculaire freinaient le recours aux soins d'ophtalmologie.

**Conclusion:**

La mise en œuvre de programmes d'ophtalmologie en milieu scolaire peut permettre de réduire sensiblement la morbidité oculaire et les retards de développement dus à des déficiences visuelles et à la cécité chez les enfants. Il est crucial de bénéficier d'un soutien politique, tout en tentant de combattre les idées fausses et la stigmatisation chez les enfants et leurs parents, pour maintenir l'accès à ces services.

## Resumen

**Objetivo:**

Revisar las intervenciones para mejorar los servicios de atención oftalmológica para los niños en edad escolar en países con ingresos entre bajos y medios.

**Métodos:**

Se realizaron búsquedas en bases de datos en línea (CINAHL, Embase®, ERIC, MEDLINE®, ProQuest, PubMed® y Web of Science^TM^) para encontrar artículos publicados entre enero de 2000 y mayo de 2018. Los estudios admisibles evaluaron la ejecución de los programas de atención oftalmológica en las escuelas e informaron de los resultados en cuanto a las tasas de cumplimiento, la calidad de los exámenes de evaluación o los cambios de actitud. Se consideró que los estudios no eran admisibles si no incluían datos de seguimiento. Dos autores seleccionaron los títulos, los resúmenes y los artículos de texto completo, y se extrajeron los datos de los artículos admisibles mediante el marco conceptual basado en derechos de disponibilidad, accesibilidad, aceptabilidad y calidad.

**Resultados:**

De las 24 559 publicaciones examinadas, 48 artículos de 13 países cumplieron los criterios de inclusión. Los factores que intervienen en el éxito de las intervenciones de atención oftalmológica en las escuelas incluyen la comunicación entre los servicios de salud y las escuelas, la disposición de las escuelas a programar el tiempo suficiente y el apoyo de los directores, el personal y los padres. Varios estudios descubrieron que, cuando el número de especialistas en atención oftalmológica es insuficiente, la formación de los profesores en la evaluación de la visión permite la prestación de un servicio de buena calidad y rentable. Además del coste de las gafas, las dificultades para obtener atención oftalmológica incluyen la alfabetización deficiente, los conceptos erróneos y la falta de conocimientos sobre la salud ocular entre los padres.

**Conclusión:**

La provisión de programas de atención oftalmológica en las escuelas tiene un gran potencial para reducir la morbilidad ocular y los retrasos en el desarrollo causados por el deterioro de la visión y la ceguera infantiles. Para mantener el acceso, es fundamental contar con apoyo basado en políticas, al tiempo que se intentan reducir los conceptos erróneos y el estigma entre los niños y sus padres.

## ملخص

**الغرض:**

مراجعة التدخلات الهادفة لتحسين خدمات العناية بالعيون لأطفال المدارس في البلدان منخفضة ومتوسطة الدخل.

**الطريقة:**

لقد ****بحثنا في قواعد البيانات على الإنترنت (CINAHL، وEmbase®، وERIC، وMEDLINE®، وProQuest، وPubMed®، وWeb of Science ^TM ^) عن المقالات المنشورة بين يناير 2000 ومايو 2018. قامت الدراسات المؤهلة بتقييم كيفية تنفيذ برامج العناية بالعيون في المدارس، وأعدت تقارير عن النتائج من حيث معدلات الامتثال، أو جودة الفحص، أو تغيرات الموقف. واعتبرنا الدراسات غير مؤهلة إذا لم يرد فيها أي ذكر لبيانات المتابعة. قام اثنان من المؤلفين بفحص العناوين والملخصات والنصوص الكاملة للمقالات، وقمنا باستخلاص البيانات من النصوص الكاملة للمقالات المؤهلة، وذلك باستخدام إطار عمل لمفاهيم توافر هذه البيانات، وإمكانية الاطلاع عليها، وحقوق الجودة.

**النتائج:**

من إجمالي 24 559 مطبوعة، كانت 48 مقالة من 13 بلداً، هي التي تفي بمعايير الاشتمال. وشملت العوامل المتبعة في التوفير الناجح لتدخلات العناية بالعيون في المدارس: الاتصال بين مقدمي الخدمات الصحية والمدارس، واستعداد المدارس لجدولة وقت كاف لهذا الغرض، ودعم المدراء وفرق العمل وأولياء الأمور. اكتشفت العديد من الدراسات أنه في حالة عدم كفاية عدد أخصائيي العناية بالعيون، فإن تدريب المعلمين على فحص الرؤية يمكنه توفير خدمة جيدة النوعية وفعالة من حيث التكلفة. وبالإضافة إلى تكلفة النظارات، فإن عوائق العناية بالعيون شملت شبه الأمية، والمفاهيم الخاطئة، ونقص المعرفة بصحة العيون بين الآباء والأمهات.

**الاستنتاج:**

يمنح تقديم برامج العناية بالعيون في المدرسة، إمكانيات كبيرة للحد من حدوث أمراض العيون، والتأخر في النمو الناجم عن ضعف البصر والعمى لدى الأطفال. بينما يحاول الدعم القائم على السياسات، الحد من المفاهيم الخاطئة ووصمة العار بين الأطفال وأولياء أمورهم، إلا أنه أيضاً أمر حاسم لاستمرار الحصول على الخدمة. ****

## 摘要

**目的:**

综述改善中低收入国家学校学生眼部护理服务的干预措施。

**方法:**

我们在线上数据库（CINAHL、Embase®、ERIC、MEDLINE®、ProQuest、PubMed® 和 Web of Science^™^）中搜索了自 2000 年 1 月至 2018 年 5 月之间发表的文章。符合入选标准的研究评估了学校开展的眼部护理课程，从依从率、筛查质量或态度改变几个方面报告结果。如果没有上报后续数据，我们就认为研究不符合入选标准。两个作者筛选出标题、摘要和全文，然后我们利用基于可用性、可得性、可接受性和质量权利的概念框架，从符合入选标准的全文中提取数据。

**结果:**

从 24,559 个出版物中筛选出满足入选标准的来自 13 个国家的 48 篇文章。学校成功提供眼部护理干预措施所涉及的因素包括卫生服务与学校之间的沟通、学校安排充足时间的意愿以及校长、学校工作人员和父母的支持。有几项研究发现，在眼部护理专家人手不够时，视力筛查的培训老师能够提供高质量且经济有效的服务。除了眼镜的费用，阻碍寻求眼部护理的因素还包括父母读写能力差、误解和缺乏眼部健康知识。

**结论:**

学校提供眼部护理课程对减少眼部发病率和缓解由童年视力损伤和失明导致的发育迟滞现象有巨大的潜在作用。在试图减少孩子和父母对此误解和污名化的同时，政策支持对继续获取服务至关重要。****

## Резюме

**Цель:**

Провести обзор мер, направленных на повышение качества оказания медицинской помощи школьникам в странах с низким и средним уровнем дохода.

**Методы:**

Авторы выполнили поиск в онлайн-базах данных (CINAHL, Embase®, ERIC, MEDLINE®, ProQuest, PubMed® и Web of Science^TM^) по статьям, опубликованным в период с января 2000 года по май 2018 года. В удовлетворяющих критериям отбора исследованиях проводилась оценка реализации программ офтальмологической помощи на базе школ, результатов отчетности с точки зрения степени приверженности, качества скрининга или изменения отношения. Мы исключили исследования, в которых не сообщалось о проведении последующего наблюдения. Два автора тщательно проверили заголовки, выдержки и полнотекстовые статьи, и из подходящих полнотекстовых статей мы извлекли данные, пользуясь рамочными критериями приемлемости, доступности и качества.

**Результаты:**

Из 24 559 публикаций критериям включения соответствовали 48 статей из 13 стран. Факторы, связанные с успешным проведением офтальмологических вмешательств на базе школ, включали обмен информацией между службами здравоохранения и школами, готовность школ выделить на это достаточное время, а также поддержку руководства, персонала и родителей. В нескольких исследованиях было установлено, что при нехватке специалистов-офтальмологов обучение учителей проведению проверки зрения позволяет обеспечить качественное и экономически эффективное обслуживание. Помимо стоимости очков, препятствия для обращения к офтальмологу включали низкий уровень грамотности, неверные представления и отсутствие знаний о здоровье глаз у родителей.

**Вывод:**

Предоставление школьных программ офтальмологической помощи имеет большой потенциал для сокращения заболеваемости органов зрения и частоты случаев задержки в развитии, вызванной нарушением зрения и слепотой у детей. Поддержка на основе политики, а также усилия по преодолению неверных представлений и социального отторжения среди детей и их родителей имеют решающее значение для постоянного доступа к медицинской помощи.

## Introduction

Vision impairment and blindness in children can have negative consequences on their health, education and prospects,^1^^–^^4^ which in turn can affect the nation’s broader economic prosperity.^5^^,^^6^ Globally, an estimated 19 million children are blind or vision impaired,^7^ with the majority of vision impairment being preventable or treatable.^8^ The highest burden of blindness is experienced by children in low-income countries, where the prevalence is estimated to be 0.9 per 1000 children compared with 0.7 per 1000 and 0.4 per 1000 children in middle- and high-income countries,^9^ respectively; this suggests there are fewer services or else increased barriers to accessing services in low-income countries.^8^

School-based eye-care interventions have the potential to provide high-quality and cost–effective services^10^ that allow the early detection of eye diseases and prevention of blindness, particularly for children living in remote locations.^11^ Identifying methods of improving and strengthening school-based eye-care interventions, particularly in low- and middle-income countries, is therefore important.

We conducted a systematic review and qualitative analysis to identify and understand methods by which eye-care services for schoolchildren in low- and middle-income countries could be improved. Our analysis was guided by the availability, accessibility, acceptability and quality conceptual framework as presented in the United Nations Committee on Economic, Social and Cultural Rights, General Comment No. 14, The Right to the Highest Attainable Standard of Health.^12^

## Methods

### Systematic search

We registered our search on the International prospective register of systematic reviews (CRD42018090316) and followed the Preferred Reporting Items for Systematic Reviews and Meta-Analyses guidelines^13^ when identifying studies assessing interventions that improve schoolchildren’s access to eye-care services.

This review set out to include all studies evaluating the impact of school-based eye-care interventions in countries that were categorized as low- and middle-income countries in 2017.^14^ Eligible studies were those that: (i) evaluated the delivery of a school-based eye-care programme through vision screening, refractive services or health promotion activities; (ii) reported the evaluation results as either spectacle compliance rates, quality of vision screening processes, quality of vision screening personnel, or changes in knowledge or attitudes due to health promotion; or (iii) provided other quantitative or qualitative results from follow-up evaluations of school-based eye-care interventions. We included cross-sectional epidemiological surveys, prospective observational studies, qualitative studies, economic evaluations and randomized controlled trials.

Studies were excluded if: (i) they were not conducted in low-and middle-income countries; (ii) the described intervention did not include schoolchildren; or (iii) they did not report data from follow-up evaluation. We also excluded meeting abstracts, conference papers, editorial discussions, books, theses and studies without primary data collection. Systematic reviews that we detected in the initial search were screened to identify any studies initially missed; they were not included in the analysis, however.

We searched the online databases CINAHL, Embase®, ERIC, MEDLINE®, ProQuest, PubMed® and Web of Science^TM^ for articles published between January 2000 and May 2018, using the search terms in Box 1. No language restrictions were placed on the search, but since search terms were in English we only retrieved English abstracts. We imported citations into Covidence software (Veritas Health Innovation, Melbourne, Australia), where two authors independently reviewed titles and abstracts. If the article could not be excluded based on abstract or title, it was included for full-text review. Two authors independently reviewed the full text of potential articles. Some article abstracts identified for full-text review did not have a full text in English, and were translated in full by a native speaker of the language.

Box 1 **Search terms used for systematic review of eye-care services for schoolchildren in low- and middle-income countries**

1. Eye-care services
2. (eye care OR blindness OR ocular OR optom* OR ophthal* OR refractive error OR myop* OR visual acuit* OR vision screening* OR visual impairment OR vision impairment OR eye-care OR vision care OR visually impair* OR amblyop* OR cataract* OR spectacle* OR eyeglass* OR glasses OR vision)
3. Education sector
4. (school* OR health education OR service* OR outreach OR school-based OR student*)
5. Children
6. (child* OR boy* OR girl* OR minor* OR adolescen* OR juvenile* OR teen* OR youth* OR parent* OR guardian* OR student*)
7. Accessibility
8. (access* OR utiliz* OR utilis* OR availability OR appropriat* OR acceptab* OR approach* OR adequ* OR inadequate OR equity OR inequity OR capability OR health seeking OR health care seeking OR social determinant* OR health literacy OR health beliefs OR barrier* OR facilitator* OR enabler* OR adherence OR compliance* OR afford* OR willingness OR knowledge OR perception* OR attitude* OR socioeconomic* OR participat* OR predictor* OR parental education OR key informant*)

Disagreements regarding inclusion or exclusion at either the title and abstract screening or full text review were resolved by discussion with a third reviewer. Two independent reviewers then appraised study quality using the Mixed Methods Appraisal Tool (v-2011, McGill University, Montreal, Canada),^15^ resolving discrepancies through discussion. We selected the appraisal tool as it has been used extensively in prior systematic reviews,^16^ and allows for the critical appraisal of qualitative, quantitative and/or mixed methods studies. This tool is preferable to the use of multiple tools, which may not allow for inter-study comparisons.

### Analysis

We analyzed the extracted data qualitatively using NVivo 11 (QSR International, Melbourne, Australia). Thematic deductive coding^17^^,^^18^ was applied to identify the a priori themes from the availability, accessibility, acceptability and quality conceptual framework.^12^ This framework applies a rights-based approach to analyzing factors related to health system coverage and accessibility, and the underlying determinants that shape them. The framework identifies the systemic characteristics that inhibit or facilitate equitable eye-care outcomes for schoolchildren, while also considering determinants related to sex, culture, education and discrimination.

## Results

**Fig. 1:**
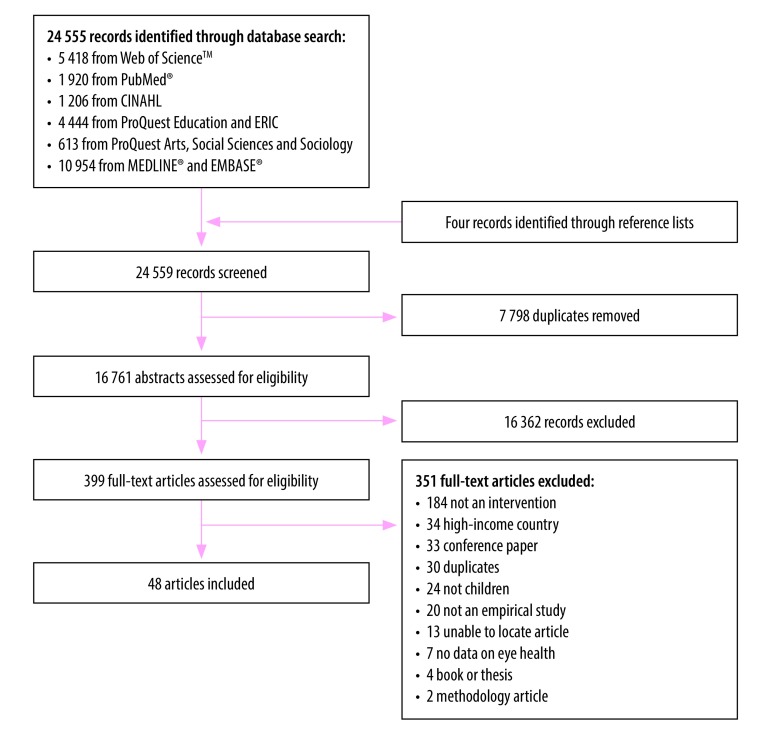
Flowchart used for the systematic review of eye-care services for schoolchildren in low- and middle-income countries

Of the 24 559 articles initially captured, we identified 48 describing school-based eye-care interventions (Fig. 1).^19^^–^^66^ Identified articles were from 13 countries spanning five World Health Organization (WHO) Regions including Africa (eight studies), the Americas (10 studies), the Eastern Mediterranean (one study), South-East Asia (18 studies) and the Western Pacific (11 studies). Identified articles comprised 19 school-based eye-care programme evaluations,^19^^–^^33^^,^^62^^–^^64^^,^^66^ 16 studies investigating spectacle compliance associations,^34^^–^^48^^,^^65^ eight studies exploring the quality of various eye-care screening personnel,^49^^–^^56^ four studies evaluating the effectiveness of eye health promotion,^57^^–^^60^ and one study that included both spectacle compliance and the quality of screening personnel.^61^ When appraised for quality,^15^ we classified 18 studies as being of high quality, 20 as medium quality and 10 as low quality. Study characteristics are outlined in Table 1 (available at: http://www.who.int/bulletin/volumes/96/10/18-212332).

**Table 1:** Studies identified in the systematic review of interventions to improve eye-care services for schoolchildren in low- and middle-income countries

Study	Country	Study design	Study sample	Purpose	Quality appraisal^a^
Castanon Holgui et al., 2006^34^	Mexico	Prospective observational	493 primary and secondary schoolchildren aged 5–18 years	Assess spectacle compliance	Low
Carvalho et al., 2007^20^	Brazil	Cross-sectional (prospective)	1517 elementary school teachers or principals	Assess teacher perceptions of school visual health campaigns	Low
Esteso et al., 2007^26^	Mexico	Prospective observational	96 primary and secondary schoolchildren (mean age 12 years)	Assess the impact of spectacles on self-reported vision health	Medium
Congdon et al., 2008^35^	South Africa	Prospective observational	8520 primary and secondary schoolchildren aged 6–19 years	Evaluate refractive error cut-offs for spectacle provision to more effectively identify children with improved vision and increase compliance	Low
Lewallen et al., 2008^58^	United Republic of Tanzania	Mixed methods	20 schools (10 intervention, 10 control), 1396 schoolchildren (grades 3 and 4)	Evaluate trachoma education outcomes, including knowledge and hygiene practices	High
Li et al., 2008^45^	China	Prospective cohort	1892 secondary schoolchildren aged 13–16 years	Assess the determinants of spectacle compliance	Medium
Odedra et al., 2008^48^	United Republic of Tanzania	Mixed methods	108 secondary school students (average age 15 years); 58 intervention group, 50 control group	Assess reasons for poor compliance following in-school provision of spectacles	Medium
Wedner et al., 2008^38^	United Republic of Tanzania	Randomized controlled trial	125 secondary schoolchildren aged 11–19 years	Assess compliance of free spectacles	High
Khandekar et al., 2009^51^	Islamic Republic of Iran	Mixed methods	15 parents and 15 teachers	Evaluate school vision screening in kindergarten, including cost and validity of teacher use	Medium
Tabansi et al., 2009^55^	Nigeria	Cross-sectional (prospective)	130 teachers, 1300 primary schoolchildren aged 6–11 years	Assess accuracy of teacher screenings, compared with research team/doctors	High
Zeng et al., 2009^40^	China	Randomized controlled trial	743 secondary schoolchildren aged 12–15 years	Evaluate children’s vision and satisfaction with ready-made spectacles	Medium
Keay et al., 2010^44^	China	Prospective observational	428 secondary schoolchildren aged 12–15 years	Determine what influences ready-made and custom-made spectacle compliance	High
Adhikari & Shrestha, 2011^49^	Nepal	Cross-sectional (prospective)	20 certified medical assistants	Assess reliability of certified medical assistants in school-based vision screening, compared with paediatric ophthalmologists	Medium
Congdon et al., 2011^65^	China	Randomized controlled trial	11 423 primary and secondary schoolchildren aged 12–17 years	Effectiveness of an educational intervention to promote spectacle purchase	Low
Noma et al., 2011^32^	Brazil	Cross-sectional (prospective)	767 parents	Determine reasons for non-adherence to ophthalmic examinations following school screening	Low
Santos et al., 2011^37^	Brazil	Cross-sectional (prospective)	62 primary schoolchildren aged 6–11 years with refractive error	Assess compliance of children to their first pair of glasses	Low
Noma et al., 2012^33^	Brazil	Cross-sectional (prospective)	14 651 primary schoolchildren aged 7–10 years	Determine reasons for non-adherence to ophthalmic examinations following school screening	High
Pereira et al., 2012^21^	Timor-Leste	Cross-sectional (prospective)	21 school health nurses, 1819 children screened	Evaluate efficacy of eye health outreach services	Medium
Rajaraman et al., 2012^23^	India	Mixed methods	52 children aged 9–17 years, 35 school staff, 13 school health counsellors, 4 parents and 3 clinicians	Evaluate the effectiveness of delivery of school health promotion by lay school health counsellors	High
Rustagi et al., 2012^36^	India	Mixed methods	51 secondary schoolchildren aged 11–18 years, sampled for refraction, out of 1075 screened	Assess the magnitude of vision impairment among children and their spectacle compliance	Medium
Balasubramaniam et al., 2013^64^	India	Qualitative	35 parents with school-aged children and 16 eye-care specialists	Effectiveness of school vision screening	Medium
Gogate et al., 2013^43^	India	Cross-sectional (prospective)	1018 secondary schoolchildren aged 8–16 years	Assess spectacle compliance among rural children	High
Rewri et al., 2013^61^	India	Cross-sectional (prospective)	7411 secondary schoolchildren aged 11–19 years	Evaluate students’ ability to self-examine their vision and seek intervention such as spectacles	High
Thummalapalli et al., 2013^60^	India	Prospective observational	104 primary school teachers	Evaluate effectiveness of eye health promotion and screening intervention among teachers	Low
Bai et al., 2014^62^	China	Cross-sectional (retrospective)	19 977 primary school students (in grades 4 and 5)	Effectiveness of school vision screening	Medium
Latorre-Arteaga et al., 2014^52^	Peru	Cross-sectional (prospective)	21 teachers	Evaluate the effectiveness of teacher vision screening and estimate childhood refractive error prevalence	Medium
Ma et al., 2014^46^	China	Randomized controlled trial	3177 primary schoolchildren aged 8–13 years in 251 schools	Assess the effect of free spectacle provision on academic performance	Medium
Puri et al., 2014^22^	India	Cross-sectional (prospective)	5404 children aged 8–15 years screened and 71 teachers surveyed	Evaluate school vision programme	Medium
Teerawattananon et al., 2014^56^	Thailand	Mixed methods	5885 students; 1335 pre-primary children aged 4–6 years, 4550 primary children aged 7–12 years	Assess accuracy and feasibility of teacher screening	Medium
Zhou et al., 2014^25^	China	Mixed methods	136 urban primary schoolchildren aged 9–11 years, 290 rural secondary schoolchildren aged 11–17 years, 16 parents	Assess the take-up of adjustable-lens spectacles among children and parents	High
Anuradha & Ramani, 2015^63^	India	Cross-sectional (prospective)	123 optometrists or optometry students	Effectiveness of optometry students in conducting school-based single-day vision screening	High
Fontenele et al., 2015^27^	Brazil	Cross-sectional (prospective)	94 school health nurses aged 20–29 years	Assess the involvement of nurses in children’s eye health	Medium
Hobday et al., 2015^28^	Timor-Leste	Mixed methods	384 primary schoolchildren aged 10–17 years; teachers and parents (number undisclosed)	Evaluate an in-school health promotional intervention	Medium
Juggernath & Knight, 2015^29^	South Africa	Randomized controlled trial	37 teachers or principals; 19 in intervention group (aged 23–67 years), 18 in control group (aged 21–59 years)	Assess teacher visual acuity screening following training	Medium
Ma et al., 2015^31^	China	Randomized controlled trial	2840 primary schoolchildren aged 8–13 years in 249 schools	Assess the safety of spectacles in rural context where a fear that spectacles harm the eyes is an important barrier	High
Priya et al., 2015^53^	India	Case–control	917 teachers	Assess cost and effectiveness of screening programme involving all teachers, compared with using a limited number of teachers	High
Saxena et al., 2015^54^	India	Cross-sectional (prospective)	40 teachers, 9838 primary schoolchildren aged 6–15 years	Assess accuracy of teacher screenings, compared with primary eye-care workers	High
Wang et al., 2015^24^	China	Cross-sectional (prospective)	4376 primary schoolchildren aged ~9–12 years; 4225 migrant children and 151 local children	Measure prevalence of spectacle need and ownership among migrant children	Low
Yi et al., 2015^39^	China	Randomized controlled trial	693 primary schoolchildren aged 10–12 years	Assess the effect of the provision of free spectacles, combined with teacher incentives, on compliance	High
Glewwe et al., 2016^42^	China	Mixed quantitative	28 798 primary schoolchildren aged 10–12 years	Determine the impact of free spectacle provision on children’s academic performance	High
Kaur et al., 2016^50^	India	Cross-sectional (prospective)	253 teachers	Assess the effectiveness of teacher screening in identifying eye problems in children	Medium
Latorre-Arteaga et al., 2016^30^	Peru	Cross-sectional (prospective)	355 teachers	Assess teacher screening programme implementation following pilot phase	High
Chan et al., 2017^57^	United Republic of Tanzania	Cross-sectional (prospective)	120 schoolchildren aged 11–12 years	Effectiveness of child-to-child health promotion strategy	High
de Melo et al., 2017^19^	Brazil	Cross-sectional (prospective)	74 primary and secondary schoolchildren aged 13–18 years	Effectiveness of an educational intervention on the topic of disability	Low
Morjaria et al., 2017^47^	India	Randomized controlled trial	460 secondary school aged 11–15 years; 232 ready-made spectacles, 228 custom-made spectacles	Compare compliance between ready- and custom-made spectacles	Medium
Paudel et al., 2017^59^	Viet Nam	Prospective cohort	300 children aged 12–15 years	Assess the effect of eye health promotion on eye health literacy in schools	High
Ebeigbe, 2018^66^	Nigeria	Qualitative	35 parents of schoolchildren aged 5–12 years	Assess the factors that influence the seeking of eye-care	Medium
Narayanan & Ramani, 2018^41^	India	Non-randomized controlled trial	8442 secondary schoolchildren aged 13–17 years screened; 238 required spectacles, of which 124 formed the intervention group and 114 the control group	Assess spectacle and referral compliance following school screening programme	Low

^a^ We appraised the quality of study methods by using the Mixed Methods Appraisal Tool (v-2011). Studies were classified as high quality if > 90% of criteria were adequate, medium quality if > 60 to 90% of criteria were adequate, low quality if > 30 to 60% of criteria were adequate and very low quality if ≤ 30% criteria were adequate. No studies of very low quality were eligible for inclusion, so no studies were excluded based on this quality assessment.

### Availability

#### School-based eye-care interventions

Recent guidelines for school-based eye-care recommend screening all primary schoolchildren for reduced visual acuity, with annual screening thereafter for new students and those previously prescribed spectacles to maintain correct prescriptions.^67^ The same guidelines are recommended for secondary schoolchildren in the first two years, followed by a re-screening of all students in the third year.^67^ However, several studies noted that these guidelines were not being met by some school-based eye-care programmes,^27^^,^^49^^,^^55^^,^^62^ with some children having never been screened.^52^ Routine vision screening within schools can provide a solution to poor uptake of care external to education systems.^33^^,^^37^ A study that conducted mass vision screenings in 51 Indian schools at the start of each academic year was identified as a cost–effective intervention.^63^ Many studies noted the availability of uncomplicated referral pathways between education and health systems, and clarity regarding referral processes, as being crucial in successful follow-up, provision of spectacles and continuity of care.^20^^,^^22^^,^^23^^,^^29^^,^^32^^,^^33^^,^^41^^,^^51^^,^^61^^,^^64^ For example, a study exploring children as potential vision screeners found that, despite being effective screeners, they were not held with sufficient credibility when referring those they screened to other health services.^57^ Inappropriate or overprescribing of spectacles was identified in studies from India and Mexico,^26^^,^^43^ suggesting that the prescribing of spectacles for moderate vision impairment should be balanced with cost and willingness to pay.^43^ Overarching factors in the provision of successful school-based eye-care interventions included communication between health services and schools, the willingness of schools to schedule sufficient time while minimizing impact,^52^^,^^63^ and the support of principals, staff and parents.^23^^,^^28^^,^^29^

#### School-based eye-care resources

An insufficient number of eye-care specialists created barriers to referrals and follow-ups in China,^62^ India^22^^,^^23^^,^^64^ and Peru.^30^ As the availability of eye-care specialists can be limited in school settings, particularly in low- and middle-income countries, studies have investigated the use of teachers, nurses, certified medical assistants and key informants for the provision of screening and basic eye-care for children.^20^^,^^21^^,^^29^^,^^51^^–^^54^^,^^56^^,^^58^^,^^60^^,^^62^ Several studies found that training teachers in vision screening enabled the provision of a good-quality^51^^,^^53^^,^^56^ and cost–effective service,^53^ while facilitating the opportunity to motivate spectacle use among students.^54^ Two studies reported that the use of teachers as vision screeners did not create significant burdens on normal workloads, and in fact enhanced rapport with children and parents.^50^^,^^56^ Evidence from Brazil,^27^ Nepal^49^ and Timor-Leste^21^ highlighted the benefits of school-based vision screening performed by trained nurses or certified medical assistants.

Studies reported that the lack of facilities^20^^,^^23^ and tools,^51^^,^^55^ such as appropriate charts for vision screening, was a potential barrier to implementing school-based eye-care programmes. The supply of low-cost spectacles was identified as increasing spectacle acceptance in China,^24^^,^^39^^,^^45^^,^^46^ Mexico^26^ and Timor-Leste.^21^ However, other studies reported that spectacle acceptance may be low with free or low-cost spectacles,^38^^,^^42^^,^^43^ which can be linked to parental concerns of poor quality.^65^

#### Health plans and policies

A key policy-based facilitator to the prioritization of child eye-care is uptake and execution of a national eye-care plan,^50^^,^^53^^,^^61^ and the inclusion of eye-care in school health policy.^28^^,^^29^^,^^50^^,^^55^^,^^58^ Studies assessing the feasibility of school-based eye-care interventions, such as the targeting of trachoma in the United Republic of Tanzania,^58^ vision screening in Peru,^30^ South Africa^29^ and Thailand,^56^ and the provision of free spectacles in China,^39^ noted that success was dependent on multidisciplinary support from health and education ministries. The level of collaboration between ministries may either facilitate^20^^,^^28^^,^^30^^,^^58^ or inhibit^62^ the coordination and success of interventions at the school level. An example from a trachoma intervention in Tanzanian schools outlined that, while elimination of trachoma was prioritized in health policies, it also needed to be incorporated into education curriculums if progress was to be made.^58^ Since achieving shared responsibility of the monitoring and execution of policies targeting eye health is considered important in the success of school-based eye-care interventions in low- and middle-income countries, partnerships between ministries and nongovernmental or private organizations are considered crucial.^21^^,^^30^^,^^52^^,^^56^^,^^62^

### Accessibility

#### Economic and physical accessibility

The cost of spectacles for children was identified as a significant barrier in many settings.^42^^,^^45^^,^^66^ Factors associated with a higher willingness to pay for spectacles included previous or current ownership of spectacles,^35^ regular spectacle wear,^45^ a recognized need for spectacles or an understanding that vision improves with spectacles.^35^^,^^56^ An additional economic factor that was reported to influence the demand of parents or guardians for eye-care services and spectacles was the loss of daily wages^64^^,^^66^ due to a lack of a carer to accompany children to additional appointments.^33^ Approaches to reduce programme costs were reported as sourcing instrumentation from local tertiary institutions,^63^ and the use of cost–effective personnel (e.g. school health counsellors^23^ or teachers^54^) and appropriate spectacle correction protocols. Examples of correction protocols include only prescribing spectacles for moderate or severe refractive error,^34^^,^^56^ and the use of ready-made spectacles.^40^^,^^47^ The geographical inaccessibility of specialist eye-care services was also a barrier reported by parents.^32^^,^^48^

#### Information accessibility

Studies have identified misconceptions regarding the causes^42^^,^^48^^,^^56^^,^^61^^,^^64^^,^^66^ and treatment^36^^,^^38^^,^^45^^,^^48^^,^^65^^,^^66^ of eye disease and vision impairment as a significant barrier. Poor literacy, lack of awareness of eye health and misconceptions among parents were all reported as having an impact on seeking care, age of presentation and treatment choices for children.^28^^,^^56^^,^^64^ A prominent misunderstanding regarding the wearing of spectacles is that they weaken or harm the eyes, resulting in the reluctance of parents to obtain them.^36^^,^^45^^,^^48^^,^^65^^,^^66^

#### Gender inequity

Gender inequity in some countries presents barriers to school attendance among girls, which can subsequently affect access to school-based eye-care. A Nepalese study reported that irregular school attendance among girls may affect access to eye-care.^49^ However, a programme providing outreach eye-care to schools in Timor-Leste resulted in greater gender equity among participants.^21^

### Acceptability

#### Cultural appropriateness

The perspectives of children, parents, eye-care specialists, teachers and the broader community all affect the success of school-based eye-care interventions. Any intervention must be culturally appropriate, as longstanding cultural practices can have a stronger influence than national health policy.^60^ For instance, a study from India identified how children’s participation in school-based eye-care programmes can be influenced by elderly family members, hindering parental decision-making.^64^ The planning of school-based vision screening should also account for religious or cultural practices,^63^ and understand emergent local beliefs. For example, a health promotion intervention in the United Republic of Tanzania was hindered by local beliefs that the services provided were linked to the recruitment of cult group followers through the outreach activities.^57^

#### Sex

Sex-related factors associated with spectacle wear varied. In India, aesthetic norms that view spectacles as cosmetically unappealing among girls^64^ were also linked to marriageability, therefore affecting uptake and utilization.^36^ Similarly, girls were more likely to refuse spectacles than boys in western China.^42^ However, studies evaluating spectacle compliance at unannounced follow-up visits found that boys were significantly less likely to be wearing spectacles (Table 2) in China^44^^,^^65^ and South Africa;^35^ no differences between boys and girls were observed elsewhere, however. Sex also influenced the success of health promotion activities in the United Republic of Tanzania^57^ and Viet Nam.^59^

**Table 2:** Spectacle compliance and acceptability from systematic review of eye-care services for schoolchildren in low- and middle-income countries

Study	Study sample and follow-up period	No. of participants (%)	Spectacle compliance or acceptance	Factors assessed for association with increased spectacle compliance or acceptance	Reasons reported for non-purchase or non-wear
**Randomized/non-randomized controlled trials: spectacle promotion**					
Congdon et al., 2011^65^	Spectacle promotion:^a^ *n* = 2236; Control: *n* = 2212 Announced single visit 6 months after dispensing spectacles	Spectacle promotion: 1622 (72.5); Control: 1578 (71.3)	Spectacle promotion: purchased, 25.7% (417/1622); wearing/in possession, 82.0% (342/417) Control: purchased, 34.0% (537/1578); wearing/in possession, 87.2% (468/537)	Purchasing spectacles:^c^ Significant: female, poorer uncorrected VA at baseline, higher refractive error, shorter follow-up after spectacle provision Non-significant: age, best corrected VA, having spectacles at baseline, randomized to intervention group Wearing/in possession of spectacles:^c^ Significant: female, poorer uncorrected VA at baseline Non-significant: age, best corrected VA, refractive error magnitude, having spectacles at baseline, randomized to intervention group	Lack of perceived need (34.0%, 738/2170), satisfied with current spectacles (30.5%, 662/2170), fears that spectacles will harm eyes (13.2%, 287/2170)
Narayanan & Ramani, 2018^41^	Intervention package:^b^ *n* = 124; Control: *n* = 114 Three unannounced single visits at 1 and 4 months after dispensing spectacles	Intervention package: 1 month, 101 (81.4); 4 months, 104 (83.9) Control: 1 month, 102 (89.5); 4 months, 96 (84.2)	Intervention package: wear at 1 month, 46.5% (47/101^g^); wear at 4 months, 52.9% (55/104^g^) Control: wear at 1 month, 17.6% (18/102); wear at 4 months, 23% (22/96)	NR	NR
**Randomized/non-randomized controlled trials: free spectacles versus purchased spectacles**					
Wedner et al., 2008^38^	Free spectacles: *n* = 68; Prescription only: *n* = 57 Single visit 3 months after intervention provided	Free spectacles: 58 (85.3); Prescription only: 50 (87.7)	Free spectacles: wearing or in possession, 46.6% (27/58) Prescription only: wearing or in possession, 26.0% (13/50)	Significant:^c^ worse VA, myopia (refractive error status) Non-significant:^c^ provided with free spectacles	NR
Ma et al., 2014^46^	Free spectacles: *n* = 527; Free spectacles + education: *n* = 626; Voucher: *n* = 492; Voucher + education: *n* = 496; Control: *n* = 510; Control + education: *n* = 526 Unannounced single visit 8 months after intervention provided	Free spectacles: 506 (96.0); Free spectacles + education: 598 (95.5); Voucher: 473 (96.1); Voucher + education: 474 (95.6); Control: 490 (96.1); Control + education: 513 (97.5)	Free spectacles: 36.8% wearing (194/527); Free spectacles + education: 43.9% wearing (275/626); Voucher: 37.6% wearing (185/492); Voucher + education: 35.4% wearing (176/496); Control: 25.3% wearing (129/510); Control + education: 26.0% wearing (137/526)	Significant:^c^ provided spectacles voucher (without education); provided spectacles (with education); provided free spectacles (without education); provided free spectacles (with education)	NR
Yi et al., 2015^39^	Free spectacles + teacher incentive: *n* = 358; Prescription + parent letter: *n* = 370 Unannounced visits at 6 weeks and 6 months	6 week follow-up: Free spectacles + teacher incentive: 352 (98.3); Prescription + parent letter: 363 (98.1) 6 month follow-up: Free spectacles + teacher incentive: 341 (95.3); Prescription + parent letter: 352 (95.1)	Free spectacles + teacher incentive: 6 weeks wearing 81.5% (287/352); 6 months wearing, 68.3% (233/341) Prescription + parent letter: 6 weeks wearing, 16.5% (60/363); 6 months wearing, 23.9% (84/352)	At the 6-month visit Significant:^c^ intervention group VA < 6/18, at least one parent wears spectacles, having spectacles at baseline Non-significant:^c^ sex, age, location, parents education, being only child, believes wearing spectacles harms vision, mathematics score, parents employed, family wealth, blackboard use	NR
**Randomized/non-randomized controlled trials: ready- versus custom-made spectacles**					
Zeng et al., 2009^40^	Ready-made: *n* = 250; custom-made: *n* = 245 Unannounced single visit 1 month after spectacles dispensed	Ready-made: 208 (83.2); custom-made: 206 (84.1)	Wearing: ready-made: 46.9% (98/209); custom-made: 51.4% (106/206)	Non-significant:^c^ being provided with custom- compared with ready-made spectacles	NR
Morjaria et al., 2017^47^	Ready-made: *n* = 232; custom-made: *n* = 228 Unannounced single visit 3–4 months after intervention provided	Ready-made: 184 (79.3); custom-made: 178 (78.1)	Wearing or had them at school: ready-made: 75.5% (139/184); custom-made: 73.6% (131/178)	Non-significant:^e^ being provided with custom- compared with ready-made spectacles	NR
**Observational studies following school eye-care programmes**					
Castanon Holguin et al., 2006^34^	*n* = 654 Single visit at 4–18 months after dispensing spectacles	493 (75.4) Participants aged ≥ 19 years were excluded	Wearing: 13.4% (66/493); in possession: 34.3% (169/493)	Significant: ^c^ older age, rural residence, mother’s education, myopia < –1.25 D, hyperopia > +0.50 D Non-significant:^c^ sex, length of time since spectacles dispensed	Concerns about appearance and being teased (16.6%, 82/493), forgot (16.6%, 82/493); use only occasionally (14.2%, 70/493)
Congdon et al., 2008^35^	*n* = 810 Unannounced single visit at 4–11 months after dispensing spectacles	483 (59.6)	Wearing: 30.8% (149/483); in possession: 13.9% (67/483)	Significant: ^c^ female, shorter time to follow-up Non-significant: age, presenting VA worse eye	NR
Li et al., 2008^45^	*n* = 674 Single visit 3 months after intervention provided	597 (88.6)	Purchased: 35.2% (210/597); wearing: 63.9% (of those that purchased; 134/210)	Significant:^c^ worse VA at baseline, spherical equivalent < –2.00 D, willing to pay more for spectacles Non-significant:^c^ age, sex, parents’ education, baseline visual field score, home floor space per resident	Owned spectacles at baseline: current spectacles are good enough (77.9%, 109/140), spectacles too expensive (11.4%, 16/140), have symptoms from current spectacles (6.4%, 9/140) Did not own spectacles at baseline: spectacles not needed (48.7%, 110/226), price (17.7%, 40/226), harmful effects of spectacles on vision (12.8%, 29/226)
Odedra et al., 2008^48^	*n* = not stated Unannounced single visit 3 months after intervention	108	Wearing: 37.0% (40/108)	Non-significant:^c^ sex	Name-calling, concerns over safety of spectacles (harm), cost
Keay et al., 2010^44^	*n* = 428 Unannounced single visit 1 month after dispensing spectacles	415 (97.0)	Wearing: 46.5% (193/415); in possession: 2.7% (11/415)	Significant:^c^ female, lower income, spectacles VA < 6/6, pupil size ≥ 4 mm, less trouble with appearance Non-significant:^c^ age, having custom-made spectacles	NR
Santos et al., 2011^37^	*n* = 79 Single visit 3 months after dispensing spectacles	62 (78.5)	Wearing: 87.1% (54/62)	Non-significant:^e^ sex, age, presenting VA	Poor frame to face adjustment, prejudice from colleagues
Rustagi et al., 2012^36^	*n* = 51 Single visit 8 months after dispensing spectacles	48 (94.1)	Purchased: 70.8% (34/48); wearing: 20.8% (10/48)	Non-significant:^e^ sex	Harmful effect of spectacles on vision (57.9%, 22/38); anticipation of teasing from other students (52.6%, 20/38); difficulty in getting married (50.0%, 19/38)
Gogate et al., 2013^43^	*n* = 2312 Unannounced single visit at 6–12 months after dispensing spectacles	1018 (44.0)	Wearing: 29.5% (300/1018); in possession: 2.1% of those not wearing spectacles (15/718)^g^	Significant:^c,f^ myopia < –2.00 D, VA < 6/18 to 3/60 at baseline, higher academic performance Non-significant:^c,f^ sex, age, VA 6/12 to 6/18	Teased about spectacles (19.8%, 142/718), spectacles broken (17.4%, 125/718), spectacles at home (16.3%, 117/718)
Rewri et al., 2013^61^	*n* = 742 Single visit 9 weeks after second vision screening	493 (66.4)	Purchased: 40.2% (198/493); wearing: 81.3% (of those who purchased) (161/198)	NR	NR
Glewwe et al., 2016^42^	*n* = 1978 Assessed acceptance of receiving spectacles rather than wearing	NR	Accepted spectacles: 70.0% (1384/1978)	Significant:^d^ male, worse VA, household head is a teacher, higher township per capita income Non-significant: having spectacles at baseline, household head is a village leader, head years of schooling, test scores, county location, Tibetan, school level	Household head refused (31.5%, 187/594), child refused (15.0%, 89/594), cannot adjust to spectacles (10.3%, 61/594)

D: dioptre; NR: not reported; VA: visual acuity.

^a^ Spectacles were recommended to be purchased after provision of a prescription, but not provided.

^b^ Intervention package consisted of 23 components related to spectacle frame and fit, education and motivation, and conduct of the screening.

^c^ Multivariate analysis.

^d^ Probit estimate associated with accepting spectacles.

^e^ Univariate analysis.

^f^ Myopia sample only.

^g^ Percentages have been recalculated as discrepancies existed between the reported values and reported percentages. Compliance rates may not be reliable.

#### Spectacle compliance

There were 17 studies either assessing interventions to increase spectacle purchase or compliance or investigating factors associated with increased spectacle wear (Table 2). While an intervention designed to promote spectacle purchase was deemed ineffective in China,^65^ one that included free spectacles was shown to increase spectacle wear in India.^41^ Free spectacles also resulted in higher compliance compared with provision of a prescription only,^38^ a prescription and a letter to the parents,^39^ or when provided in conjunction with an education programme.^46^ In observational studies following school-based eye-care programmes, spectacle compliance ranged from 13.4% (66/493) in Mexico^34^ to 87.1% (54/62) in Brazil.^37^

Due to variations in reporting, it was not possible to identify which demographic factors were consistently associated with spectacle purchase and ongoing wear. However, girls, children with higher maternal education and children with poorer visual acuity at presentation were generally more likely to be wearing spectacles at follow-up. Many studies^28^^,^^33^^,^^36^^,^^37^^,^^41^^,^^43^^,^^45^^,^^48^^,^^56^^,^^64^^,^^66^ reported that children’s experiences or perspectives of wearing spectacles were linked to stigma and discrimination, or included verbal^36^^,^^37^^,^^48^^,^^56^ or physical abuse.^43^ In Timor-Leste, 18.1% (43/237) of children surveyed believed that vision-impaired people could not attend school.^28^ Other common reasons for not wearing prescribed spectacles included a lack of perceived need,^45^^,^^65^^,^^66^ fear of potential harm,^36^^,^^45^^,^^48^^,^^65^^,^^66^ affordability^45^^,^^48^^,^^66^ and parental objections.^42^^,^^66^ Support networks within schools, including health promotion interventions,^28^^,^^41^^,^^59^ teacher training^60^ and curriculum-based eye health education,^23^^,^^58^ were key in correcting negative perceptions regarding spectacles or eye care.

### Quality

#### Spectacle quality and provision guidelines

Concerns about the quality of spectacles^34^^,^^43^^,^^65^ or the inability to replace them^42^^,^^48^ were reported as factors related to the unwillingness to purchase or wear them. Ready-made spectacles are often a cost–effective and acceptable strategy for reducing the time of a clinician’s visit and to dispense spectacles,^40^^,^^47^ and were acceptable to many children.^39^^,^^44^^,^^47^

As poorer visual acuity has been associated with increased spectacle acceptance and compliance, several studies have recommended only prescribing spectacles to children with more severe refractive error.^34^^,^^47^^,^^54^^,^^56^ However, a randomized controlled trial investigating the effect of various refractive cut-off protocols on compliance found no associations.^35^ As small differences in refractive cut-offs are likely to have a significant impact on spectacle provision and programme costs, further investigations of spectacle prescribing guidelines are warranted.

#### Quality of trained teachers as screeners

There are inherent benefits in using teachers to conduct initial screening as compared with visiting eye-care specialists, particularly in terms of cost–effectiveness. Identified studies reported on the sensitivity (the percentage of children correctly identified with vision impairment) and specificity (the percentage of children correctly identified as not having vision impairment) of various school personnel (Table 3). While teachers have demonstrated adequate sensitivity and specificity in a variety of settings, sensitivity was reduced with younger children^56^ and when screening cut-off thresholds were lower.^54^^,^^55^ The type of vision chart used was also suggested to affect teacher sensitivity and screening function.^55^ Screening specificity is also critical due to the unnecessary burden placed on the limited numbers of eye-care specialists. One study reported that teachers sometimes overestimate the risk, and refer children who do not require visual correction.^20^

**Table 3:** Ability of various cadres to identify vision impairment from review of school-based eye-care programmes in low- and middle-income countries

Study	Screening cadre	Population screened	Gold standard	Outcome	Percentage sensitivity (95% CI)	Percentage specificity (95% CI)	PPV (95% CI)	NPV (95% CI)
Khandekar et al., 2009^51^	Kindergarten teachers	7768 children aged 3–6 years	Optometrist	VA < 20/40 (6/12) correctable by spectacles of > ± 0.5 D	74.5 (72.7–76.3)	97.2 (96.7–97.6)	96.6	86.6
Tabansi et al., 2009^55^	Teachers	1300 children aged 6–11 years	Study investigators and doctors	VA < 6/18 in either or both eyes	53.3	98.4	79.3	94.7
Adhikari & Shrestha, 2011^49^	Certified medical assistants	528 children aged 3–7 years	Paediatric ophthalmologist	VA < 6/12 (HOTV^a^ chart)	80.0	99.0	–	–
				Abnormal red reflex test	16.0	97.0	–	–
				Screening pass/fail	58.0	96.0	30.4	98.8
Rewri et al., 2013^61^	7411 children aged 10–19 years	817 children with self-assessed impaired vision aged 10–19 years	Optometrist	VA ≤ 6/12 (self-examination)	96.2 (94.5–97.4)	90.2 (87.8–92.2)	90.8 (88.6–92.7)	96.0 (94.1–97.2)
Latorre-Arteaga et al., 2014^52^	Teachers	33 children aged 3–5 years	Ophthalmic assistants	VA < 6/9 in one or both eyes	–	95.8 (92.8–98.7)	59.1 (36.3–81.9)	–
		30 children aged 5–11 years)		VA ≤ 6/9 in one or both eyes	–	93.0 (89.0–96.9)	47.8 (25.2–70.4)	–
Teerawattananon et al., 2014^56^	Pre-primary teachers	1132 children, pre-primary grades	Ophthalmologist	Presenting VA < 20/40 (< 6/12) (‘E’ chart)	25.0 (23.0–27.0)	98.0 (97.0–99.0)	–	–
	Primary teachers	4171 children, primary grades		Presenting VA < 20/40 (6/12) (Snellen chart)	59.0 (57.0–61.0)	98.0	–	–
Priya et al., 2015^53^	Selected teachers	6225 children aged 6–17 years	Ophthalmic team	VA < 20/30 (6/9.5) in either eye	–^ b^	–^ b^	–^ b^	–^ b^
	All teachers	3806 children aged 6–17 years		VA < 20/30 (6/9.5) in either eye	–^ b^	–^ b^	–^ b^	–^ b^
Saxena et al., 2015^54^	Teachers	9383 children aged 6–15 years	Primary eye-care worker	VA < 6/9.5	79.2 (77.0–81.2)	93.3 (92.7–93.8)	–	–
				VA < 6/12	77.0 (74.1–79.7)	97.1 (96.7–97.4)	–	–
				VA < 6/15	55.0 (54.1–59.7)	99.1 (98.8–99.2)	–	–
Kaur et al., 2016^50^	Teachers	129 children aged ≤ 16 years	Ophthalmologists	VA < 6/9 in either eye	98.0 (88.0–99.9)^c^	27.8 (18.6–37.2)^c^	46.2 (36.6–56.1)^c^	95.7 (76.0–99.8)^c^

D: dioptre; PPV: positive predictive value; NPV: negative predictive value; VA: visual acuity.

^a^ An HOTV vision testing chart contains the letters H, O, T and V.

^b^ Sensitivity and specificity results not provided; studies are included in the table for completeness.

^c ^Sensitivity, specificity and 95% confidence intervals were calculated from reported values of true positive, true negative, false positive and false negative.

Training can improve teacher performance, as highlighted in examples from Peru where strategies to increase teacher engagement resulted in higher levels of teacher involvement and increased confidence in vision screening.^30^^,^^52^ Elsewhere, strategies used to increase teacher screening quality and engagement included: involving all class teachers in the vision screening programme, as compared with selected teachers;^53^ using adequate and structured training to increase knowledge and screening performance;^29^^,^^58^ involving ophthalmologists in training to increase motivation;^53^ and greater emphasis on accurately measuring visual acuity.^56^ Financial incentives may encourage teachers to participate,^51^^,^^56^ and were shown to increase spectacle compliance through additional teacher motivation.^39^

## Discussion

This systematic review revealed many factors that affect the delivery of eye-care services to children in schools. The rights-based framework^12^ allowed us to explore the various dimensions of service delivery, extending beyond physical availability to accessibility, acceptability and service quality. The consideration of culture, discrimination and economic factors highlights the importance of social and systemic inequality and its impact on accessibility.^68^^,^^69^ Our review explored how school-based eye-care services function and connect with general health systems, how stakeholders interact with school-based eye-care services and programmes, and the possible paths to meeting population needs in a way that is equitable and responsive.^70^^,^^71^ School-based eye-care interventions (including vision screenings) are key to reducing morbidity and developmental delays associated with vision impairment, while promoting early detection and prevention of eye diseases.^67^^,^^72^ Increasing the availability of school-based eye-care interventions in low- and middle-income countries can help to address the burden on poorly resourced secondary and tertiary eye-care,^73^^,^^74^ and enhance access for underserved rural children.^21^^,^^30^

Effective coordination between education and health systems is essential for appropriate referral pathways and follow-up mechanisms.^22^^,^^33^^,^^34^^,^^52^ At the policy level this requires cooperation between the ministries of health and education, and a national eye-care plan that includes school-based eye-care.^52^^,^^56^^,^^58^ Without a policy-based foundation, programmes to provide high-quality and cost–effective school-based eye-care, including training teachers^29^^,^^50^^,^^52^^,^^58^ and school nurses^21^ in vision screening, will face challenges in acquiring resources and achieving sustainable outcomes.

Recent standard guidelines for comprehensive school-based eye-care programmes state that vision screening should use only one row of optotypes at the 6/9 visual acuity level.^67^ Standardized assessment and equipment (using a tumbling E chart) would reduce the current inconsistency in referral standards, and allow improved monitoring of quality and compliance. We also identified teacher training strategies that could be applied to increase teacher engagement and the quality of screening.

Because economic considerations are important in low- and middle-income countries, the provision of low-cost or free spectacles can improve access. However, the cost–effectiveness of screening and prescribed spectacles must be carefully considered to ensure sustainability. Our review identified the need to improve perceptions and awareness of eye-care services and treatments (particularly spectacles) among parents and children; we suggest health promotions that aim to (i) reduce misconceptions and stigma among parents, children and the broader community; and (ii) engage potential school-based eye-care providers such as teachers, school nurses and community health workers. A rights-based approach focusing on the link between good vision and childhood educational development is recommended, while also considering cultural factors.

Our systematic review was executed according to recommended guidelines.^13^ The literature consisted of a broad range of qualitative and quantitative studies, and our use of the rights-based conceptual framework^12^ enabled us to analyze the data in a well structured manner. However, data extraction and coding was only performed by a single reviewer due to time and resource constraints, which may have resulted in the omission of some data.

In conclusion, providing school-based eye-care interventions is challenging and reliant on economical, sociocultural, geographical and policy-based factors. With these determinants considered, school-based eye-care interventions have great potential to reduce the morbidity and developmental delays caused by childhood vision impairment and blindness. Teachers and nurses are well placed to provide school vision screenings, particularly where there is a lack of eye-care specialists. Policy-based support, with a focus on health systems rather than a focus on a single disease, is crucial for school-based eye-care interventions to be sustainable.
